# Revamping Medical Education in India: New Guidelines for Eligibility Qualifications of Medical Faculty

**DOI:** 10.7759/cureus.38925

**Published:** 2023-05-12

**Authors:** Rajmohan Seetharaman

**Affiliations:** 1 Pharmacology and Therapeutics, Seth Gordhandas Sunderdas Medical College (GSMC) and the King Edward Memorial (KEM) Hospital, Mumbai, IND

**Keywords:** credible journals, medical education technology, indexing databases, research collaboration, publication standards, promotion requirements, faculty regulations, national medical commission (nmc)

## Abstract

The National Medical Commission (NMC) in India has introduced new guidelines for the eligibility qualifications of faculty in medical institutions in 2022, aimed at enhancing the quality of medical education and healthcare in the country. The guidelines include an increased minimum publication requirement for promotion to professorship, the consideration of various types of publications, and mandatory courses in biomedical research and medical education technology. The guidelines also recommend reputable indexing databases and journals to improve the quality of research work. The NMC's efforts are expected to promote research collaboration, evidence-based clinical practice, and consistent teaching standards. However, it is essential to ensure that the recommended databases and journals are legitimate and credible. Overall, the NMC's initiatives to improve medical education in India are commendable, and it is hoped that they will lead to significant improvements in the quality of healthcare in the country.

## Editorial

The National Medical Commission (NMC) is a regulatory body that is committed to reforming the medical education system in India by introducing significant changes in the curriculum and regulations for medical faculty. The NMC is a proactive organization that continuously works toward enhancing the quality of medical education and healthcare in the country. Its latest guidelines, issued in 2022, are a part of this ongoing effort to raise the standard of medical education in India [1].

One of the significant changes is the increased minimum publication requirements for promotion to professorship. According to the new guidelines, a minimum of four publications is required for the position of professor, with at least two publications during the tenure of an associate professorship. Moreover, two publications are mandatory for promotion to the position of associate professor. The guidelines do not specify the order of authorship to be considered for promotion, which encourages authors to engage in more collaborative research. This approach may result in a higher quality of research work and a more holistic view of the research findings [1]. The higher standards for promotion incentivize faculty members to publish more research and engage in more collaborative work, which can ultimately lead to higher-quality research. By requiring a minimum number of publications for promotions, the guidelines ensure that faculty members are producing a substantial body of work that contributes to their field and demonstrates their expertise. Furthermore, the fact that the guidelines do not specify the order of authorship for promotion also encourages collaboration and joint authorship among researchers. This collaborative approach can lead to a more holistic view of research findings, as well as foster new and innovative ideas, leading to a higher quality of research [2].

The promotion will only be granted based on original research papers, meta-analyses, case series, and systematic reviews, which is in line with the previous guidelines. Additionally, the recommended indexing databases include Medline, PubMed Central, Science Citation Index, Expanded Embase, Scopus, and the Directory of Open Access Journals [1]. These databases are considered reputable and are expected to improve the quality of research work. The revisions made to the guidelines can help revive research skills and improve publication standards. By providing a list of recommended indexing databases, the NMC has simplified the process of choosing reputable journals. However, it is crucial to scrutinize the legitimacy and credentials of the journals to avoid falling victim to predatory journals. Predatory journals do not have a rigorous peer-review process and can harm the credibility of the research work published in them [3].

The NMC has emphasized the significance of fundamental courses in biomedical research and medical education technology as a requirement for promotion [1]. These courses provide valuable insights into the latest developments in medical research and education technology, which can help medical faculty improve their teaching methods and facilitate the transfer of knowledge to students. The NMC has recognized the importance of these courses and made them mandatory for teachers to qualify for promotion. However, due to the limited availability of slots for the basic course in medical education technology, the NMC has recently amended the guidelines to allow the faculty to complete the course within three years of the promotion date [1]. This amendment was made to enable more teachers to complete the course and acquire the necessary skills and knowledge to improve medical education standards in the country. The basic course in medical education technology covers a wide range of topics, including teaching and learning methodologies, curriculum development, assessment and evaluation, and instructional design. It provides an overview of various technological tools that can be used to improve the teaching and learning process, such as learning management systems, virtual learning environments, and multimedia resources. By completing this course, medical teachers can enhance their skills in designing and implementing effective teaching strategies that promote active learning and critical thinking among students. They can also learn how to use various technological tools to create engaging and interactive learning experiences for their students, which can lead to better retention of knowledge and skills [4].

In addition to original research papers, meta-analyses, case series, and systematic reviews, the new guidelines must also permit the consideration of review articles, case reports, and commentaries published in indexed journals of high standards for promotion as stated by Subramanian et al. [2]. These types of publications are essential for supporting evidence-based clinical practice and have an important role in disseminating information to healthcare professionals. Review articles provide a comprehensive summary of existing research on a particular topic, which can help medical teachers and practitioners stay up-to-date with the latest advancements in their fields. Case reports describe rare or unusual cases encountered in clinical practice, which can contribute to the development of new hypotheses, treatment plans, and diagnostic strategies. Commentaries offer expert opinions on various issues and can provide critical insights into important topics in medical education. By including these types of publications in the promotion requirements, the guidelines seek to enhance the proficiency of medical teachers by encouraging them to engage in a broader range of academic activities. This can ultimately contribute to the development of a more holistic and evidence-based approach to medical education, which is essential for improving healthcare outcomes in India [5].

Addressing potential challenges or barriers to implementing the new NMC guidelines and publication criteria for promotion in medical institutions across India is crucial to understanding the potential impact of the new guidelines. One potential challenge is the limited resources or infrastructure to support the increased publication requirements, mandatory courses, or the use of medical education technology. Medical institutions may struggle to provide the necessary support for faculty members to meet the new publication requirements, and there may be a shortage of resources for mandatory courses or technology. These challenges could lead to increased stress and workload for faculty members, which could ultimately impact the quality of the research produced. It is important to consider and address these potential barriers to ensure the successful implementation of the new guidelines and to achieve the desired outcomes [1].

In conclusion, the new guidelines introduced by the National Medical Commission for the eligibility qualifications of faculty in medical institutions in 2022 are a significant step toward revamping medical education in India. The guidelines are expected to improve the quality of research work and promote consistent teaching standards across medical establishments. The revision of the minimum publication requirements for professorships and the inclusion of various types of publications are likely to encourage research collaboration and support evidence-based clinical practice. However, it is essential to ensure that the recommended indexing databases and journals are legitimate and credible. Overall, the NMC's efforts to enhance medical education in India are commendable, and it is hoped that they will result in significant improvements in the quality of medical education and healthcare in the country.
